# The Impact of Knee Braces on Plantar Pressure Distribution in Elderly Individuals: Implications for Fall Risk Prevention

**DOI:** 10.3390/sports14020078

**Published:** 2026-02-11

**Authors:** José Lumini, Andrea Ribeiro, André Schneider, António M. Monteiro, João Sousa

**Affiliations:** 1 Centro Interdisciplinar em Ciências da Saúde (CICS), Instituto Superior de Saúde (ISAVE), Rua Castelo de Al-mourol nº 13, 4720-155 Amares, Portugal; jose.lumini@isave.pt (J.L.); andrea.ribeiro@isave.pt (A.R.);; 2 Centro de Investigação em Reabilitação (CIR), Escola Superior de Saúde, Instituto Politécnico do Porto, Rua Doutor António Bernardino de Almeida 400, 4200-072 Porto, Portugal; 3 Research Center in Physical Activity, Health and Leisure (CIAFEL), Faculty of Sports, University of Porto (FADEUP), 4200-450 Porto, Portugal; 4 Laboratory for Integrative and Translational Research in Population Health (ITR), University of Porto, 4200-450 Porto, Portugal; 5 Laboratory of Biomechanics of Porto (LABIOMEP-UP), Faculty of Sport, University of Porto, 4200-450 Porto, Portugal; 6 Department of Sports Sciences, Instituto Politécnico de Bragança, 5300-253 Bragança, Portugal; andrecschneider@gmail.com; 7 Research Centre for Active Living and Wellbeing (Livewell), Instituto Politécnico de Bragança, 5300-253 Bragança, Portugal; 8 Faculty of Physical Activity and Sports Sciences and Institute of Biomedicine (IBIOMED), Universidad DeLeón, 24007 León, Spain

**Keywords:** plantar pressure, knee brace, elderly, baropodometry, fall risk, proprioception

## Abstract

(1) Background: Falls are a major public health concern in older adults, largely due to age-related declines in proprioception and postural control. Although knee braces are commonly prescribed to enhance joint stability and sensory feedback, their effects on plantar pressure distribution remain unclear; (2) Methods: Thirteen community-dwelling older adults (mean age: 79.6 ± 3.2 years) participated in a repeated-measures study under three conditions: no brace, knee brace A, and knee brace B. Plantar pressure variables were assessed barefoot during quiet standing using a baropodometric platform. Conditions were compared using non-parametric Friedman tests; (3) Results: Significant differences were observed for left foot total surface area (*p* = 0.041) and left rearfoot surface area (*p* = 0.020). Compared with no brace, brace A increased plantar contact area, whereas brace B reduced it. No significant differences were found for pressure magnitude, load distribution, or right foot variables; (4) Conclusions: Knee braces induce subtle, brace-specific and lateralized changes in plantar pressure distribution, potentially reflecting altered postural control strategies. Although limited to specific variables, these effects may be clinically relevant for fall risk assessment and individualized knee brace prescription in older adults.

## 1. Introduction

Falls are a major public health problem in older adults, with an estimated 28% of community-dwelling adults aged over 65 years experiencing at least one fall per year, and an exponential increase in fall risk with advancing age [1], and falls are not just a matter of physical trauma but have a cascade of consequences, such as loss of independence, reduced quality of life, increased healthcare utilization and increased mortality [2]. Age-related declines in proprioceptive acuity are a major contributor to impaired balance and increased fall risk in older adults, alongside broader changes in muscle function, neuromuscular control, and joint integrity [3,4]. Proprioception—the ability to sense joint position and movement—provides continuous afferent input from mechanoreceptors located in muscles, tendons, ligaments, and joint capsules to the central nervous system, and plays a critical role in the detection and correction of postural sway [5].

Proprioceptive acuity declines with age [6,7], for example, knee joint position sense is over twice as inaccurate in older than younger adults, and as the quality of this sensory feedback deteriorates, it becomes more difficult to detect and correct postural sway and the risk of falls increases.

The knee joint is a key contributor to posture and gait, and age-related reductions in knee proprioception are further compromised by knee OA, a condition prevalent in older adults, because adults with knee OA have poorer proprioceptive thresholds than age-matched healthy adults [8,9], and these deficits are strongly associated with functional limitations and increased falls risk. Degradation of joint tissues (particularly mechanoreceptor location) distorts sensory feedback, impairs motor control and leads to the adoption of movement patterns that compromise stability; however, given the critical role that knee function and proprioception play in balance, considerable attention has focused on targeting these deficits [10]. Proprioceptive training exercises have been shown to improve balance, reduce pain and falls risk in older adults with knee OA [11], and in addition to exercise, external supports such as knee braces may serve to enhance knee stability and sensory feedback, because knee braces can stimulate cutaneous and joint mechanoreceptors through gentle compression and contact, and may improve joint position sense and neuromuscular control [12,13].

Preliminary evidence suggests that some knee braces may also improve lower-limb kinematics and patient-reported outcomes in people with OA [14], and one potential pathway through which knee function may affect falls risk is through its effects on plantar pressure distribution (the distribution of forces applied to the ground through the foot during standing and walking). Plantar pressure distribution represents a downstream biomechanical expression of postural control, reflecting how individuals regulate foot–ground interaction to maintain upright stance. Variables such as total contact area, regional loading (e.g., rearfoot versus forefoot), and left–right asymmetry provide insight into weight-bearing strategies, base-of-support modulation, and compensatory adjustments commonly observed in older adults during quiet standing. In this context, baropodometric measures offer an indirect but objective window into how sensory and neuromuscular factors influence postural regulation at the foot–ground interface.

Beyond the knee joint, ankle function—particularly ankle dorsiflexion—plays a critical role in postural control and fall risk in older adults, as it directly influences foot–ground interaction and compensatory balance strategies during both quiet standing and gait. Limitations in ankle dorsiflexion have been associated with altered balance responses and reduced adaptability to postural perturbations in aging populations. In parallel, recent advances in wearable and inertial sensor technologies have enabled reliable and objective assessment of ankle dorsiflexion and lower-limb kinematics in clinical and field-based settings, supporting their growing role in fall risk screening and prevention strategies. These technological developments highlight the importance of considering multisegmental sensorimotor contributions to balance control when evaluating the biomechanical effects of interventions targeting a single joint [15].

Altered plantar pressure patterns have been associated with increased fall risk in older adults, particularly in studies conducted under dynamic conditions such as gait, where changes in regional loading, pressure gradients, and asymmetry have been linked to instability and falls [15,16,17]. Although evidence from dynamic tasks predominates, quiet standing remains a relevant biomechanical context, as it represents a fundamental condition for postural regulation and allows the assessment of baseline weight-bearing strategies and foot–ground interaction in the absence of locomotor demands [16,17,18,19].

The present study aimed to investigate the effect of two different knee braces on plantar pressure distribution in older adults during static standing. Accordingly, we hypothesized that knee brace use would alter selected plantar pressure measures during quiet standing when compared with a no-brace condition.

## 2. Materials and Methods

### 2.1. Study Design and Participants

The sample was recruited from “Fisiomovement”, a community-based physiotherapy and exercise program for older adults developed by ISAVE (Instituto Superior de Saúde). Participants regularly attended group sessions, and they were familiar with the research team and assessment procedures, because they had previously interacted with them. The inclusion criteria were: age 65 years or older; able to stand unsupported for at least 10 s; and no neurological pathology that may affect postural or balance control, therefore individuals with known neurological diseases were excluded.

Parkinson’s disease, stroke or peripheral neuropathy; Lower limb injury or surgery in the last six months; People who could not perform the protocol, thus a total of 13 subjects participated in this study. All were Portuguese speaking adults, and there were both men and women, so all participants gave their written consent.

Information regarding history of falls in the previous 6–12 months, clinical diagnosis of knee osteoarthritis, use of assistive devices for ambulation, visual or vestibular impairments, and medication use potentially affecting balance was not systematically collected. Therefore, these variables were not included in the analysis and should be considered when interpreting the findings, particularly given the relatively high-functioning, community-dwelling nature of the sample.

### 2.2. Ethical Considerations

This study was conducted in accordance with the principles of the Declaration of Helsinki and followed all relevant national and institutional guidelines for research involving human participants [20]. Ethical approval was obtained from the ISAVE Ethics Committee (Approval No. 2025/05-08). All participants received verbal and written information about the objectives and procedures of the study and were given the opportunity to ask questions before participation. Written informed consent was obtained from each participant prior to data collection.

To ensure confidentiality and data protection, all personal information was anonymized and stored securely, accessible only to the research team. Participants were informed of their right to withdraw from the study at any time without penalty or impact on their involvement in the FisioMovement programme. No adverse events occurred during the study procedures.

### 2.3. Data Collection Protocol

All testing was performed during a single session for each subject and all testing was conducted in a quiet room to ensure consistency among subjects. Upon arrival, each subject completed a brief questionnaire regarding demographics such as name, age, weight, height, shoe size, dominant leg, and any existing medical conditions, and this was done so that the researchers could gather relevant information.

Each subject was tested under three conditions using a fixed-baseline design followed by a randomized brace order: (1) no brace (control), (2) knee brace A, and (3) knee brace B. The no-brace condition was consistently assessed first to establish an individual baseline under unassisted standing conditions, after which the two brace conditions were administered in a randomized order to minimize sequence effects between brace types. The absence of full counterbalancing across all conditions should be considered a potential source of order or habituation effects, particularly in the context of static balance assessment.

During each trial, subjects stood barefoot on the Baropodometric platform Freemed (from Sensor Medica, Guidonia Montecelio, Italy) with feet approximately shoulder width apart, arms at the side, and fixated on a target placed 2 m in front of them, and they were instructed to remain as still as possible throughout the 10 s trial. A trial duration of 10 s was selected in accordance with the baropodometric platform manufacturer’s recommendations and previous studies indicating that short-duration trials are sufficient to characterize plantar pressure distribution during quiet standing in older adults. Plantar pressure variables were calculated as mean values over the entire recording period, and no initial transient phase was excluded from the analysis.

Subjects were given a rest period of at least 60 s between trials to prevent fatigue, and to allow for brace application and removal, so that they could recover and prepare for the next trial.

After completion of all trials, participants were asked which knee brace they perceived as the most comfortable. This information was collected for descriptive purposes only and was not included in the statistical analysis or used to guide interpretation of the plantar pressure outcomes.

### 2.4. Statistical Analysis

All statistical analyses were performed using IBM SPSS Statistics version 30 software (IBM Corp., Armonk, NY, USA) for non-parametric repeated measures analysis. Descriptive statistics were calculated for all variables. Demographic characteristics are reported as mean and standard deviation, whereas plantar pressure variables are presented as median and interquartile range (IQR) to maintain consistency with the non-parametric analytical approach.

Given the small sample size and the ordinal or ranked nature of several plantar pressure outcomes, non-parametric statistical methods were selected a priori for all analyses. Tests of normality (Shapiro–Wilk) were conducted for descriptive purposes only and are reported for completeness [21].

The Friedman test, a non-parametric alternative to repeated measures ANOVA, was used to compare the three experimental conditions (no brace, brace A, brace B) for each plantar pressure variable [22]. The Friedman test is appropriate for analyzing repeated measurements on the same subjects when data are non-normally distributed. The test statistic (χ^2^) and associated *p*-value were calculated for each variable. Statistical significance was set at α = 0.05 (two-tailed) [22]. Effect size estimates for the Friedman tests (e.g., Kendall’s W) were not calculated in the present study. Therefore, the magnitude of the observed differences cannot be quantified, and the results should be interpreted primarily in descriptive terms. This limitation should be considered when evaluating the practical and clinical relevance of the statistically significant findings.

When the Friedman test indicated a statistically significant overall effect, no post hoc pairwise comparisons were performed. Therefore, any comparisons between individual conditions (e.g., no brace vs. brace A or no brace vs. brace B) are presented descriptively and should not be interpreted as statistically confirmed pairwise differences.

Results are reported as median values with interquartile range (IQR, 25th–75th percentile) to appropriately represent the central tendency and variability of non-normally distributed data.

Given the exploratory nature of the study and the relatively large number of plantar pressure variables examined, no formal correction for multiple comparisons was applied. Therefore, *p*-values are interpreted descriptively rather than as confirmatory evidence, and the results should be considered hypothesis-generating. The potential for inflated type I error should be taken into account when interpreting statistically significant findings.

## 3. Results

### 3.1. Participant Characteristics

Thirteen participants (N = 13) completed all study procedures. Demographic and anthropometric characteristics are presented in Table 1. The sample had a mean age of 79.62 ± 3.18 years (median 80.0 years, range 75–84 years), mean height of 156.62 ± 5.69 cm (median 156.0 cm, range 145–168 cm), and mean body weight of 76.76 ± 7.07 kg (median 75.03 kg, range 68.0–93.7 kg). Mean shoe size was 39.62 ± 2.06 (median 39.0, range 37–44).

### 3.2. Plantar Pressure Comparisons Across Conditions

The Friedman test revealed statistically significant differences between the three experimental conditions (no brace, brace A, brace B) for two left foot variables: total surface area and rearfoot surface area. Complete results for all measured variables are presented in Table 2.

### 3.3. Significant Findings

The Friedman test identified overall differences across the three experimental conditions for two left-foot variables: total surface area and rearfoot surface area. These findings should be interpreted within the context of an exploratory analysis involving multiple outcome measures, in which *p*-values are considered descriptive rather than confirmatory. No post hoc pairwise comparisons were performed; therefore, differences between individual conditions are presented descriptively and should not be interpreted as statistically confirmed pairwise effects.

Total Surface Area—Left Foot. An overall difference across conditions was detected (χ^2^ = 6.39, df = 2, *p* = 0.041). The median total surface area was 171.0 cm^2^ (IQR 34.0) in the no-brace condition, 177.0 cm^2^ (IQR 33.0) with brace A, and 160.0 cm^2^ (IQR 29.0) with brace B.

Rearfoot Surface Area—Left Foot. An overall difference across conditions was also detected for left rearfoot surface area (χ^2^ = 7.80, df = 2, *p* = 0.020). Median rearfoot surface area values were 92.0 cm^2^ (IQR 22.0) in the no-brace condition, 94.0 cm^2^ (IQR 33.0) with brace A, and 89.0 cm^2^ (IQR 22.0) with brace B.

### 3.4. Non-Significant Findings

All remaining variables assessed—including maximum pressure, average pressure, load percentage, absolute load, forefoot surface area, forefoot and rearfoot weight ratios, and all corresponding right foot parameters—showed no statistically significant differences across the three conditions (all *p* > 0.05). Notably, the right foot showed no significant differences in any measured parameter, indicating that the effect of knee bracing on plantar pressure distribution was lateralized to the left foot only.

Bilateral load distribution remained balanced across all conditions, with left foot load percentage ranging from 50 to 52% and right foot load percentage from 48 to 50%, with no significant differences between conditions (*p* = 0.867). Similarly, forefoot-rearfoot weight distribution patterns were stable across conditions, with no significant shifts in weight-bearing strategy between the anterior and posterior regions of either foot.

## 4. Discussion

The purpose of this study was to examine the influence of two types of knee braces on plantar pressure distribution in older adults during quiet standing. The initial hypothesis that knee bracing would significantly affect plantar pressure distribution was only partially supported. Overall differences across experimental conditions were identified for two plantar contact area measures of the left foot—total surface area (*p* = 0.041) and rearfoot surface area (*p* = 0.020)—while all other plantar pressure variables, including pressure magnitudes, load distribution, and all right-foot measures, did not differ across conditions.

The present findings therefore indicate brace-specific changes in plantar contact pattern measures during quiet standing, limited to selected surface area variables of the left foot. As no post hoc pairwise comparisons were performed, differences between individual conditions are presented descriptively and should not be interpreted as statistically confirmed pairwise effects. Importantly, no significant differences were observed in plantar pressure magnitude variables, indicating that the detected effects were forefoot- and rearfoot-contact related rather than reflecting changes in pressure intensity or load redistribution.

Changes in plantar contact area may arise from multiple biomechanical mechanisms, including alterations in foot posture, stance configuration, or material compliance [4,23]. However, because no measures of postural control, stability, neuromuscular activity, proprioception, or lower-limb alignment were collected, the underlying mechanisms responsible for the observed brace-specific contact area patterns cannot be determined within the present study. Thus, references describing proprioceptive, neuromuscular, or alignment-related effects of knee bracing [12,13], are discussed here only to contextualize existing hypotheses in the literature, rather than to explain the mechanisms underlying the present findings.

In contrast to brace A, brace B was associated with lower plantar contact area values during quiet standing. Beyond this descriptive observation, no inferences can be made regarding stability, restriction of motion, comfort, or altered loading strategies, as these constructs were not directly assessed.

Although previous studies have linked plantar pressure characteristics to balance performance and fall risk in dynamic contexts [16], no significant differences in plantar pressure magnitude were observed in the present study. Therefore, no inferences regarding pressure concentration or fall risk can be drawn from the current findings, and references addressing these associations are cited solely to frame the broader biomechanical literature rather than to support causal interpretation.

The left-foot specificity of the observed differences warrants consideration. Prior work has suggested that postural contributions may differ between dominant and non-dominant limbs [24], and asymmetries are common in older adults [25]. Nevertheless, limb dominance, baseline asymmetry, and bilateral functional contributions were not explicitly assessed in the present study. Fully counterbalanced designs and explicit evaluation of limb dominance and baseline asymmetry would be necessary to clarify whether the unilateral findings observed here reflect methodological, biomechanical, or individual factors.

Previous literature has suggested that knee braces and joint stabilizers may influence sensorimotor function and movement patterns under certain conditions [25,26]. However, other studies report limited or context-dependent effects, particularly in individuals without pronounced sensory or motor deficits [14,27]. The relatively healthy and functionally independent profile of the present sample may partly explain why the observed effects were modest and localized, and why changes were confined to surface area measures rather than pressure peaks or load redistribution.

These preliminary findings indicate that different knee brace designs may yield distinct plantar contact-area responses during quiet standing; however, any clinical implications require confirmation using functional, dynamic, and outcome-based measures. Accordingly, the present results should not be interpreted as supporting clinical recommendations.

Although plantar pressure asymmetry has been associated with fall risk in previous studies [28], asymmetry-related outcomes were not the primary focus of the present investigation. Future studies should therefore examine whether different knee brace designs influence plantar pressure asymmetry metrics during both static and dynamic tasks. Investigations incorporating gait, turning, stair negotiation, or responses to perturbations would provide greater insight into the functional relevance of brace-related plantar pressure changes [29]. Additionally, studies involving older adults with knee osteoarthritis, balance impairments, or a history of falls may help determine whether the modest effects observed here are amplified in more vulnerable populations.

Several limitations should be acknowledged. First, the small sample size (N = 13) limits statistical power and generalizability. Second, multiple plantar pressure variables were examined without formal adjustment for multiple comparisons, and no post hoc pairwise analyses were conducted; therefore, *p*-values should be interpreted descriptively. Third, effect size estimates were not calculated, limiting the assessment of the practical relevance of the observed differences. Fourth, each condition was assessed using a single, short-duration trial, and the reliability of these measures was not established. Collectively, these analytical constraints reinforce the exploratory nature of the findings and warrant cautious interpretation.

## 5. Conclusions

This study identified small, brace-specific changes in left-foot plantar contact area measures during quiet standing, while most plantar pressure variables did not differ across conditions. These findings highlight that different knee brace designs may elicit localized alterations in plantar contact patterns under static conditions. Given the exploratory nature of the study and the absence of functional or dynamic outcomes, the results should be interpreted cautiously. Further research incorporating larger samples and dynamic, functional measures is required to clarify the biomechanical and clinical relevance of these observations.

## Figures and Tables

**Table 1:** Descriptive characteristics of the study participants.

Characteristic	N	Mean	SD	Median	Min.	Max.
Age (years)	13	79.62	3.18	80.0	75.0	84.0
Height (cm)	13	156.62	5.69	156.0	145.0	168.0
Weight (kg)	13	76.76	7.07	75.03	68.0	93.68
Shoe size	13	39.62	2.06	39.0	37.0	44.0

SD (Standard Deviation); N (Sample).

**Table 2:** Plantar pressure variables across experimental conditions during quiet standing (median [IQR]).

Variable	No Brace Median (IQR)	Brace A Median (IQR)	Brace B Median (IQR)	χ^2^	*p*-Value
Pressure variables					
Max Pressure—Left Foot (g/cm^2^)	555 (186)	496 (85)	545 (88)	2.00	0.368
Max Pressure—Right Foot (g/cm^2^)	511 (152)	482 (71)	566 (119)	3.23	0.199
Avg Pressure—Left Foot (g/cm^2^)	233 (41)	215 (41)	226 (32)	4.31	0.116
Avg Pressure—Right Foot (g/cm^2^)	216 (50)	230 (46)	230 (40)	0.46	0.794
Surface area variables					
Total Surface—Left Foot (cm^2^)	171 (34)	177 (33)	160 (29)	6.39	0.041 *
Total Surface—Right Foot (cm^2^)	158 (33)	163 (24)	166 (19)	1.12	0.571
Forefoot Surface—Left Foot (cm^2^)	76 (31)	78 (6)	89 (19)	0.15	0.926
Forefoot Surface—Right Foot (cm^2^)	70 (21)	76 (18)	75 (18)	1.00	0.607
Rearfoot Surface—Left Foot (cm^2^)	92 (22)	94 (33)	89 (22)	7.80	0.020 *
Rearfoot Surface—Right Foot (cm^2^)	89 (21)	92 (23)	90 (28)	1.85	0.397
Load distribution variables					
Load Percentage—Left Foot (%)	52 (7)	50 (3)	52 (4)	0.29	0.867
Load Percentage—Right Foot (%)	48 (7)	50 (3)	48 (4)	0.29	0.867
Load—Left Foot (kg)	37 (8)	38 (3)	40 (5)	0.54	0.763
Load—Right Foot (kg)	34 (8)	37 (4)	35 (7)	0.54	0.763
Forefoot Weight Ratio—Left (%)	43 (7)	42 (14)	46 (11)	0.52	0.770
Forefoot Weight Ratio—Right (%)	43 (13)	44 (15)	43 (8)	0.76	0.684
Rearfoot Weight Ratio—Left (%)	57 (7)	58 (14)	54 (11)	0.52	0.770
Rearfoot Weight Ratio—Right (%)	57 (13)	56 (15)	57 (8)	0.76	0.684
Rearfoot Load—Left (%)	29 (6)	30 (9)	28 (6)	0.50	0.779
Rearfoot Load—Right (%)	27 (6)	28 (3)	27 (5)	0.55	0.761

Note: χ^2^ = Friedman test statistic. IQR = interquartile range (25th–75th percentile). * *p*-values are reported descriptively due to the exploratory nature of the analysis and the absence of correction for multiple comparisons.

## Data Availability

The data presented in this study are available on request from the corresponding author due to ethical and privacy restrictions, as the dataset contains personal and sensitive information of the participants, and public sharing could compromise participant confidentiality.
